# Integrating machine learning with bioinformatics for predicting idiopathic pulmonary fibrosis prognosis: developing an individualized clinical prediction tool

**DOI:** 10.3389/ebm.2024.10215

**Published:** 2024-12-23

**Authors:** Hongmei Ruan, Chunnian Ren

**Affiliations:** ^1^ Department of Pediatric Neurology, Chengdu Women’s and Children’s Central Hospital, School of Medicine, University of Electronic Science and Technology of China, Chengdu, China; ^2^ Department of Pediatric Surgery, Chengdu Women’s and Children’s Central Hospital, School of Medicine, University of Electronic Science and Technology of China, Chengdu, China

**Keywords:** idiopathic pulmonary fibrosis, machine learning, prediction model, random survival forest, hub gene

## Abstract

Idiopathic pulmonary fibrosis (IPF) is a chronic interstitial lung disease with a poor prognosis. Its non-specific clinical symptoms make accurate prediction of disease progression challenging. This study aimed to develop molecular-level prognostic models to personalize treatment strategies for IPF patients. Using transcriptome sequencing and clinical data from 176 IPF patients, we developed a Random Survival Forest (RSF) model through machine learning and bioinformatics techniques. The model demonstrated superior predictive accuracy and clinical utility, as shown by the concordance index (C-index), the area under the operating characteristic curve (AUC), Brief scores, and decision curve analysis (DCA) curves. Additionally, a novel prognostic staging system was introduced to stratify IPF patients into distinct risk groups, enabling individualized predictions. The model’s performance was validated using a bleomycin-induced pulmonary fibrosis mouse model. In conclusion, this study offers a new prognostic staging system and predictive tool for IPF, providing valuable insights for treatment and management.

## Impact statement

The lack of specificity of the clinical symptoms of IPF makes it difficult to predict the prognosis of IPF patients by clinical symptoms, and the establishment of a prediction model by identifying prognostic genes has become another possible method to determine the prognosis of IPF patients. To establish a prediction model with higher predictive performance, we compared the predictive performance of the conventional model and machine learning model, identified a prediction model with the best predictive performance, and developed it into a prediction tool. The current study provides a new tool for individualized treatment of IPF.

## Introduction

IPF is a chronic, progressive interstitial lung disease defined by fibrosis, inflammation, and lung structure destruction [1, 2]. Predominantly affecting the elderly and middle-aged, IPF carries a poor prognosis [3], with a median survival time of 2–4 years post-diagnosis [3]. The current therapeutic mainstays, pirfenidone and nintedanib, offer only symptomatic relief by slowing the fibrotic progression [4, 5]. Identifying effective methods to understand the disease progression and prognosis of IPF patients is crucial for clinicians to enhance the management of IPF patients and formulate individualized treatment regimens. However, the non-specific clinical symptoms of IPF, such as dyspnea and cough, overlap with many other diseases, rendering the prediction of disease progression unreliable based on respiratory function and imaging examination alone. Therefore, there is a need to explore molecular-level biomarkers and develop an accurate prognostic model tool to track and evaluate the prognosis of IPF.

Recently, machine learning-based prediction models have emerged as potential tools for disease progression prediction [6, 7]. These models are believed to better handle complex, high-dimensional data relationships and more accurately reflect the associations between variables and outcomes compared to traditional linear models such as the Cox model. However, no studies have yet established a machine learning-based prognostic model for IPF, and the comparative predictive efficacy of traditional versus machine learning models in IPF remains unclear.

Given the non-specificity clinical manifestations in patients with IPF, prediction models based on these clinical manifestations, respiratory function, and imaging examinations have shown limited predictive power. With advancements in bioinformatics, molecular-level understanding of disease prognosis has been applied across various diseases. Identifying differentially expressed genes (DEGs) in IPF patients can aid clinicians in accurately determining the individualized prognosis by selecting prediction models with superior predictive performance. Additionally, establishing a prognostic staging system based on the most accurate predictive model can more precisely identify high-risk IPF patients.

The essence of IPF, a chronic progressive interstitial lung disease, lies in the fibrotic process, which involves tissue fibrosis, epithelial cell damage, and aberrant tissue repair [8, 9]. While previous studies have highlighted the role of immune cell infiltration in fibrotic injury and repair [8–10], few have investigated the molecular mechanisms that differentiate IPF patients across various risk groups. Our study employs enrichment analysis to identify pathways specifically enriched in different risk groups of IPF patients and to explore the extent of immune cell infiltration in the high-risk groups, potentially enhancing our understanding of the molecular mechanisms in high-risk IPF populations.

## Materials and methods

### Data acquisition and normalization

The GSE70866 dataset comprises gene expression profiles and clinical data from 176 bronchoalveolar lavage cells of IPF patients. It includes gene expression data from 112 IPF patients collected using the GPL14550 platform (Agilent-028004 SurePrint G3 Human GE 8 × 60 K Microarray, Agilent Technologies) and from 64 IPF patients using the GPL17077 platform (Agilent-039494 SurePrint G3 Human GE v2 8 × 60 K Microarray). We merged the data from these two platforms using inSilicoMerging and subsequently performed batch effect removal analysis to generate a consolidated expression matrix [11]. To verify the effectiveness of the batch effect removal, we conducted Principal Component Analysis (PCA) on the dataset’s expression matrix both before and after the removal process. PCA is a dimensionality reduction technique that extracts key feature vectors from high-dimensional data, transforming it into a lower-dimensional representation and visualizing these features in 2D or 3D graphs.

### Variance analysis

The “limma” package was used to identify DEGs between 176 IPF and 20 normal samples [12]. The Benjamin-Hochberg method was used to adjust original p-values, while the false discovery rate (FDR) procedure was employed to determine fold-changes (FC). Expressions with |logFC|>1.5 and FDR<0.05 were considered significantly different. Heat maps and volcano maps were constructed to show the details of the variance analysis.

### Weighted correlation network analysis (WGCNA)

To investigate the co-expression relationships among genes and their association with phenotypes, we constructed a gene co-expression network utilizing the “WGCNA” package in R software [13]. For all calculation of pair-wise genes, Pearson’s correlation were performed. Using the TOM (Topological Overlap Matrix) model, average linkage hierarchical clustering was performed on Genes with similar expression profiles to classify them into Gene modules. Modules with a distance under 0.25 were combined, resulting in 24 co-expression modules.

### Enrichment analysis

Gene Ontology (GO) [14] analysis is a common method to perform large-scale functional enrichment studies. The Kyoto Encyclopedia of Genes and Genomes (KEGG) [15] is a widely used database that stores information about genomes, biological pathways, diseases, and drugs. The biological process enrichment of hub genes was performed by the R package clusterprofiler with input filtering criteria of p.adj <0.05 and FDR value (q.value) < 0.5 statistically significant. Gene set enrichment analysis (GSEA) is a calculation method that determines whether a set of prior defend genes show statistically significant and consistent differences between two biological states [16]. In this study, GSEA was employed to discern the biological processes and signaling pathways that varied between the high-risk and low-risk IPF groups using the R package clusterProfiler. Significance was determined with a p-value threshold of less than 0.05.

### Identification of prognostic genes

The Least Absolute Shrinkage and Selection Operator (LASSO) is a linear regression technique that incorporates shrinkage, making it suitable for survival analysis with high-dimensional data. In this study, we employed the R package glmnet, which facilitates LASSO regression analysis, to identify the most influential variables among the hub genes in our train set. Subsequently, we conducted a multivariate Cox regression analysis using the variables selected by the LASSO regression analysis.

### The development and evaluation of model

Model development are performed based on the scikit-survival module for the Python platform, including algorithm optimization and training. For the RSF model, we employed grid search for algorithm optimization and utilized the RSF algorithm within ML for model training. Grid search fine-tuned the RSF model’s hyperparameters, which included the number of estimators (10, 100, and 500), minimum of samples split (3, 5, 6, and 10), minimum of samples leaf (1, 2, 4, and 10), and maximum depth (2, 5, 10, and None). Model performance was assessed using the test set, with evaluations based on the C-index and AUC at 1, 2, and 3 years. The C-index is a widely recognized metric that quantifies the ability of a model to predict outcomes. A model with an AUC greater than 0.75 is generally considered to exhibit excellent discrimination [7]. Calibration was appraised using the Brier score at the same time points; a Brier score of 0.25 or less signifies favorable model calibration [17]. DCA was conducted to determine the clinical net benefit, a method that calculates the net benefit under a risk threshold and is primarily employed to assess the clinical utility of the model [18].

### The interpretation of model

Clinicians require a straightforward method to elucidate how the model predicts patient survival. The Shapley Additive Explanations (SHAP) plot serves as an effective tool for this purpose. This game-theoretic approach to model output interpretation reveals the contribution of each variable to the predicted outcome [19]. The SHAP plot is generated utilizing the scikit-survival module within the Python environment.

### Prognostic staging system for IPF patients

The X-tile is a bio-informatics tool utilized for biomarker assessment and optimization of outcome-based cut-points [20]. Kaplan Meier (KM) curve analysis serves as the method to analyze and infer patient survival times from the data, examining the relationship between survival times, outcomes and the influence of various factors along with their relative impact. The individual risk score, derived from the output of the RSF model, stratified IPF patients in both the train and test sets into high-risk and low-risk groups. A comparison of overall survival between these two groups was conducted using KM curve survival analysis, with the log-rank test employed for statistical testing.

### Immune function analysis

To identify immune characteristics of IPF and normal samples, as well as high-risk and low-risk groups, we analyzed their expression data using the Cell-type Identification By Estimating Relative Subsets Of RNA Transcripts (CIBERSORT) web portal^1^. The analysis was conducted iteratively 1,000 times to ascertain the relative percentages of 22 distinct immune cell types [21]. Subsequently, we compared these relative percentages across IPF and normal samples, in addition to high-risk and low-risk groups.

### Validation of the PF model

The PF mouse model was established through a single intratracheal administration of Bleomycin (BLM) at a dosage of 2 mg/kg (MCE, USA) [22]. On day 14 post-administration, lung tissues were harvested from the sacrificed mice to proceed with further experiments. Lung fibrosis severity was evaluated through Masson’s trichrome staining and Western blot analysis. Furthermore, the expression levels of hub genes integrated into the RSF model were validated using Quantitative Reverse Transcription-Polymerase Chain Reaction (qRT-PCR) assays.

### Statistical analysis

For the statistical analysis, we employed R, a programming language and software environment for statistical computing (R Foundation for Statistical Computing, Vienna, Austria), and the Sangerbox platform. Model training was conducted using Python (Version 3.10), developed by Guido van Rossum in Scotts Valley, CA, United States. Both the Cox model and the RSF model were implemented utilizing the scikit-survival module (Version 0.19.0).

## Results

### Identification of hub genes in IPF

The flow chart of the study is shown in Supplementary Figure S1 mRNA expression data obtained from the GPL14550 platform and GPL17077 platform of GSE80776 were merged and subjected to a batch effect removal analysis to obtain a combined expression matrix with 176 IPF and 20 healthy people involving 19,531 Genes. After removing the batch effect we can observe a uniform distribution of the merged matrix (Supplementary Figure S2).

A total of 4,187 DEGs were obtained by variance analysis and included 3,970 upregulated and 217 downregulated genes (Supplementary Figure S3). The DEGs were visualized by the volcano map (Supplementary Figure S3A) and heatmap (Supplementary Figure S3B).

**FIGURE 1:**
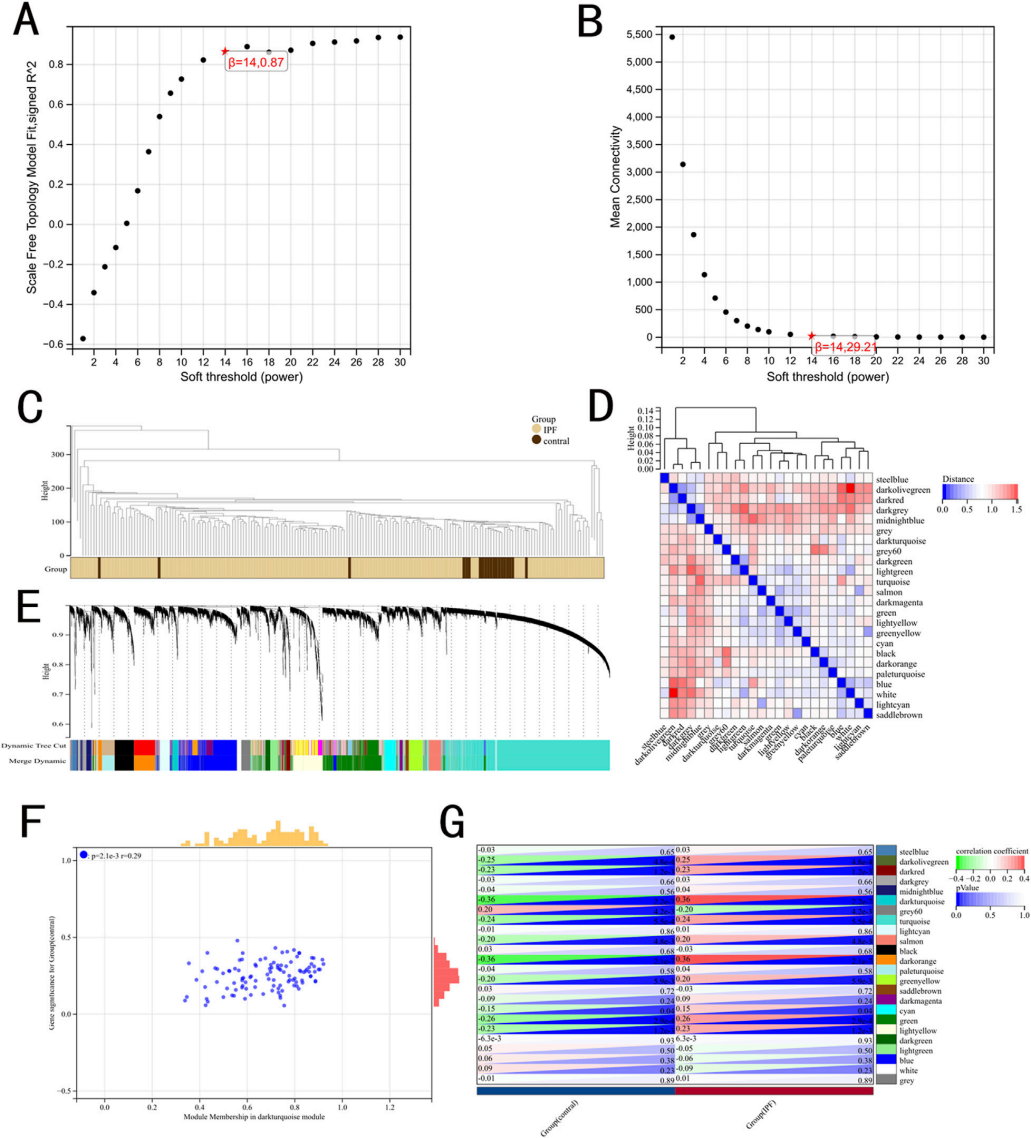
Results of the WGCNA. **(A)** The corresponding scale-free topological model fit indices at different soft threshold powers. **(B)** The corresponding mean connectivity values at different soft threshold powers. **(C)** Cluster dendrogram of samples. **(D)** Cluster dendrogram of module feature. **(E)** Cluster dendrogram of genes. **(F)** Correlations between different modules and clinical traits. **(G)** Correlation of module membership and gene significance in the darkturquoise module.

WGCNA was used to identify IPF-related hub genes. As shown in Figures 1A, B, the horizontal axis is the soft threshold and the vertical axis is the evaluation parameters of scale-free network. The higher the value of evaluation parameters, the more consistent the network is with the characteristics of scale-free network. The optimal soft-thresholding power was set as 14 with R square value of 0.87. Finally, 24 modules are identified by hierarchical clustering and optimal soft threshold capability (Figures 1C–E). The darkturquoise module, which exhibited the highest positive correlation with IPF, contained 28 genes (Figures 1F, G).

**FIGURE 2:**
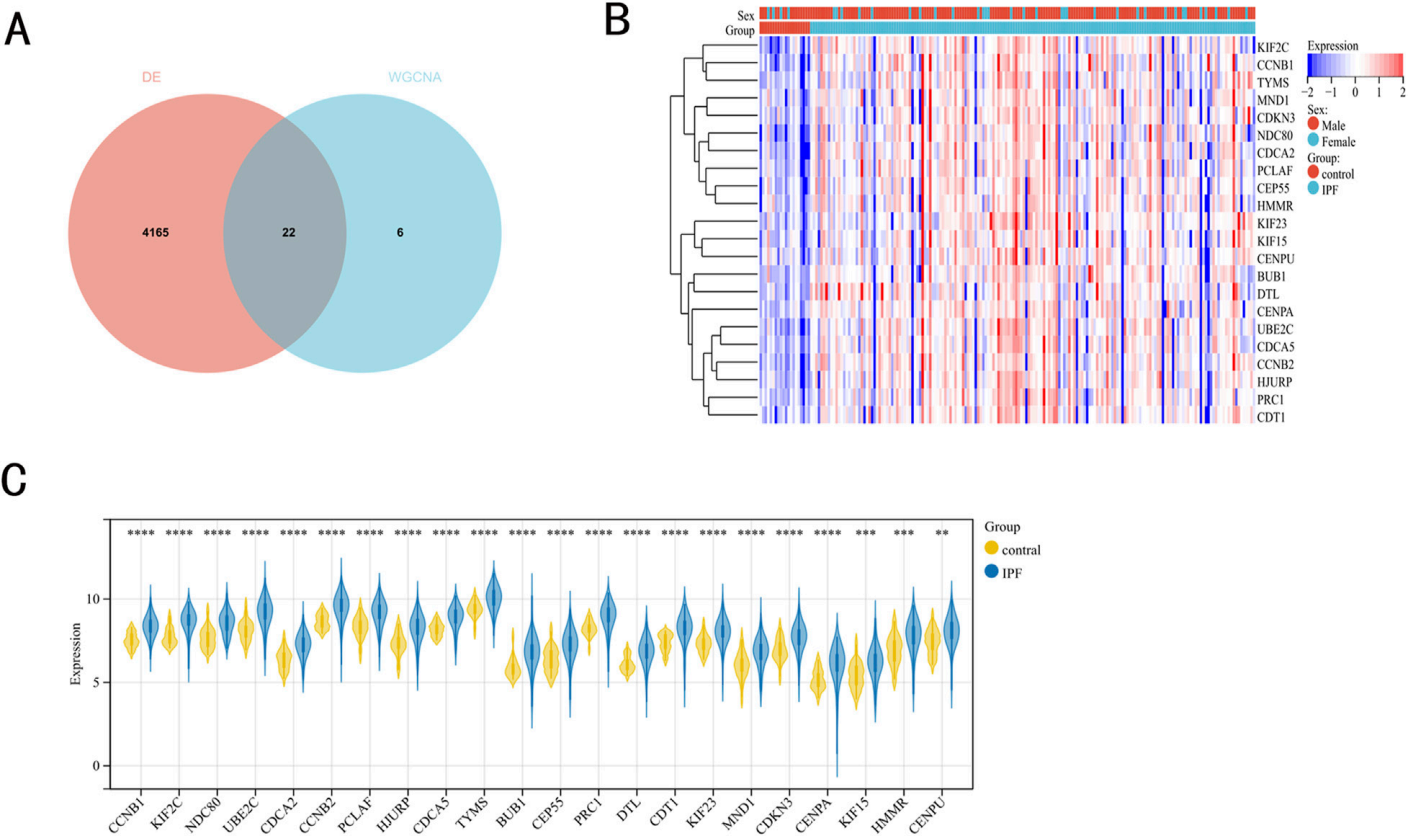
IPF-related hub genes. **(A)** 22 hub genes were obtained by taking the intersections of the DEGs, and darkturquoise module genes of the WGCNA. **(B, C)** Heatmaps and boxplots demonstrated the expression of 22 hub genes in IPF patients.

A venn diagram was utilized to identify DEGs selected in both variance analysis and WGCNA analysis. Consequently, 22 IPF-related hub genes were determined (Figure 2A). In IPF patients, heatmaps and boxplots revealed significant upregulation of these 22 genes (Figures 2B, C).

### Functional enrichment analysis

GO and KEGG pathway enrichment analyses were conducted to deepen our understanding of the functions of the identified hub genes. The analysis of KEGG revealed that these hub genes were mainly associated with cell cycle, p53 signaling pathway, FoxO signaling pathway, and Cellular senescence (Supplementary Figures S4A, B). In addition, the analysis of GO enrichment revealed that these hub genes were primarily associated with the cell cycle, mitotic cell cycle, and cell cycle process (Supplementary Figure S4C).

### Identification of prognostic genes

**FIGURE 3:**
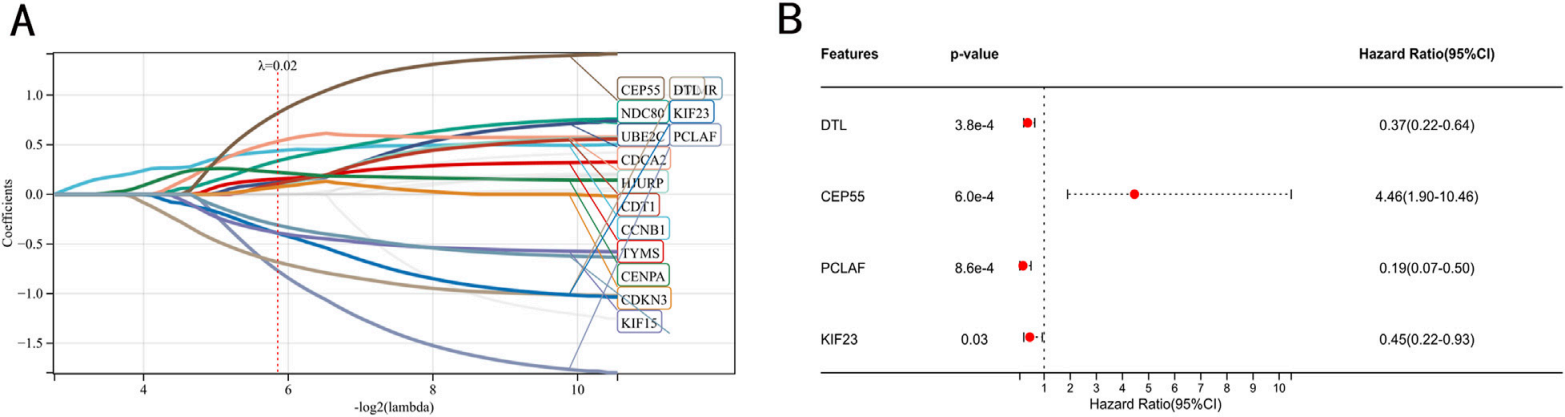
Identification of prognostic genes. **(A)** Screening of characteristic genes by LASSO regression analysis. **(B)** Multivariate Cox regression analysis to identification of prognostic genes in the train set.

As shown in Figure 3A, we conducted LASSO regression analysis on 22 IPF-related hub genes screened and further screened 14 hub genes. Subsequently, proceeded with a multivariate Cox regression analysis, which led to the identification of four significant prognostic genes. Among these, one gene was identified as a potential risk gene, while the other three were recognized as potential protective genes (Figure 3B).

### Model development and evaluation

We constructed both the Cox and RSF models using data from the train set. The RSF model’s hyperparameters were optimized through grid search, with the final configuration set as follows: 10 estimators, 5 minimum of samples split and 1 minimum of samples leaf.

**FIGURE 4:**
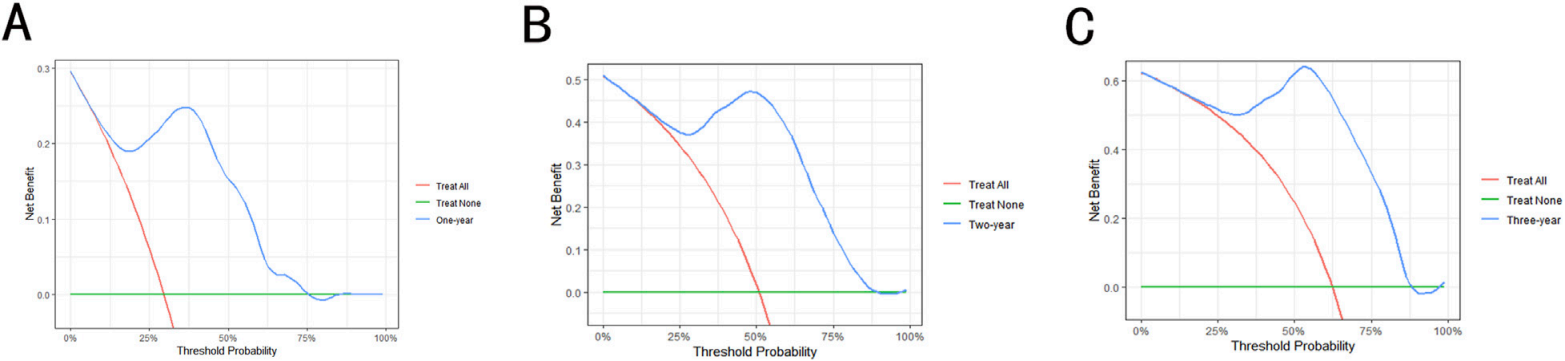
The decision analysis curves of RSF model. **(A)** The 1-year decision analysis curve of RSF model. **(B)** The 2-year decision analysis curve of RSF model. **(C)** The 3-year decision analysis curve of RSF model. In the decision analysis curve, the x-axis represented the threshold probability while the y-axis represented the clinical net benefits.

Model validation was conducted using the C-index, Brier score and AUC. The validation of the models in the test set is shown in Table 1. The results show that the C-index of RSF model is 0.840, which is obviously superior to Cox model. Similarly, the AUC (1-year, 2-year and 3-year) and Brier scores (1-year, 2-year and 3-year) also have better performance on the RSF model. Meanwhile, we used DCA to assess the potential clinical significance of the RSF model, the DCA regarding the RSF model showed fair clinical net benefits in 1, 2, and 3 years in Figure 4.

**TABLE 1:** The validation of the models in the test set.

Model	C-index	AUC			Brier score		
		1-Year	2-Year	3-Year	1-Year	2-Year	3-Year
RSF model	0.840	0.941	0.942	0.958	0.099	0.120	0.007
Cox model	0.544	0.569	0.552	0.578	0.198	0.264	0.255

RSF, random survival forest.

### Model interpretation

**FIGURE 5:**
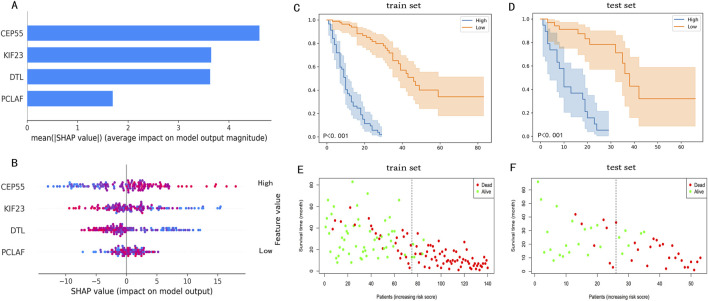
The SHAP plot of the RSF model and risk stratification of IPF patients. **(A)** A Shap plot after quantization of each variable. **(B)** A SHAP plot that includes the distribution of variables for all patients, the color of the dot symbolized the numerical value of the variable. In the SHAP plot, the length of the horizontal axis where each variable is located represents the variable’s contribution to the outcome. For example, the variable (CEP55) is the most significant risk factor. The higher the expression of CEP55, the higher the probability of a poor prognosis. **(C)** KM survival curves after risk stratification of patients in the train set. **(D)** KM survival curves after risk stratification of patients in the test set. In KM survival plots, the blue line represents the high-risk group, the orange line represents the low-risk group, and the P-value in the plots is the log-rank test result. **(E)** The survival state distribution of the train set. **(F)** The survival state distribution of the test set.

The SHAP plot in Figure 5 was used to interpret visually the global importance of variables in the RSF model, the variables in the RSF model were listed in descending order of importance. As seen in Figure 5A, the contributions of all variables to the RSF model were quantified to establish their ranking. The distribution of the scatter plot in Figure 5B represents each variable across all samples in the RSF model. Among these, CEP55 emerged as the most influential variable, succeeded by KIF23, DTL and PCLAF.

### Prognostic staging system for IPF patients

Patients within the train set were assigned scores by the RSF model, leading to the stratification of patients into high-risk (risk score>23.1) and low-risk (risk score<=23.1) groups. Meanwhile, the KM analysis and log-rank test results, which highlighted significant differences between the high-risk and low-risk groups, are displayed in Figures 5C, D which demonstrated a significant difference between the two groups. The distribution of survival states for both the train and test set is shown in Figures 5E, F. These findings indicate that the RSF model, based on four prognostic genes, can effectively predict IPF prognosis, demonstrating high accuracy in both the train and test sets.

### Application of the RSF model in individual survival prediction

**FIGURE 6:**
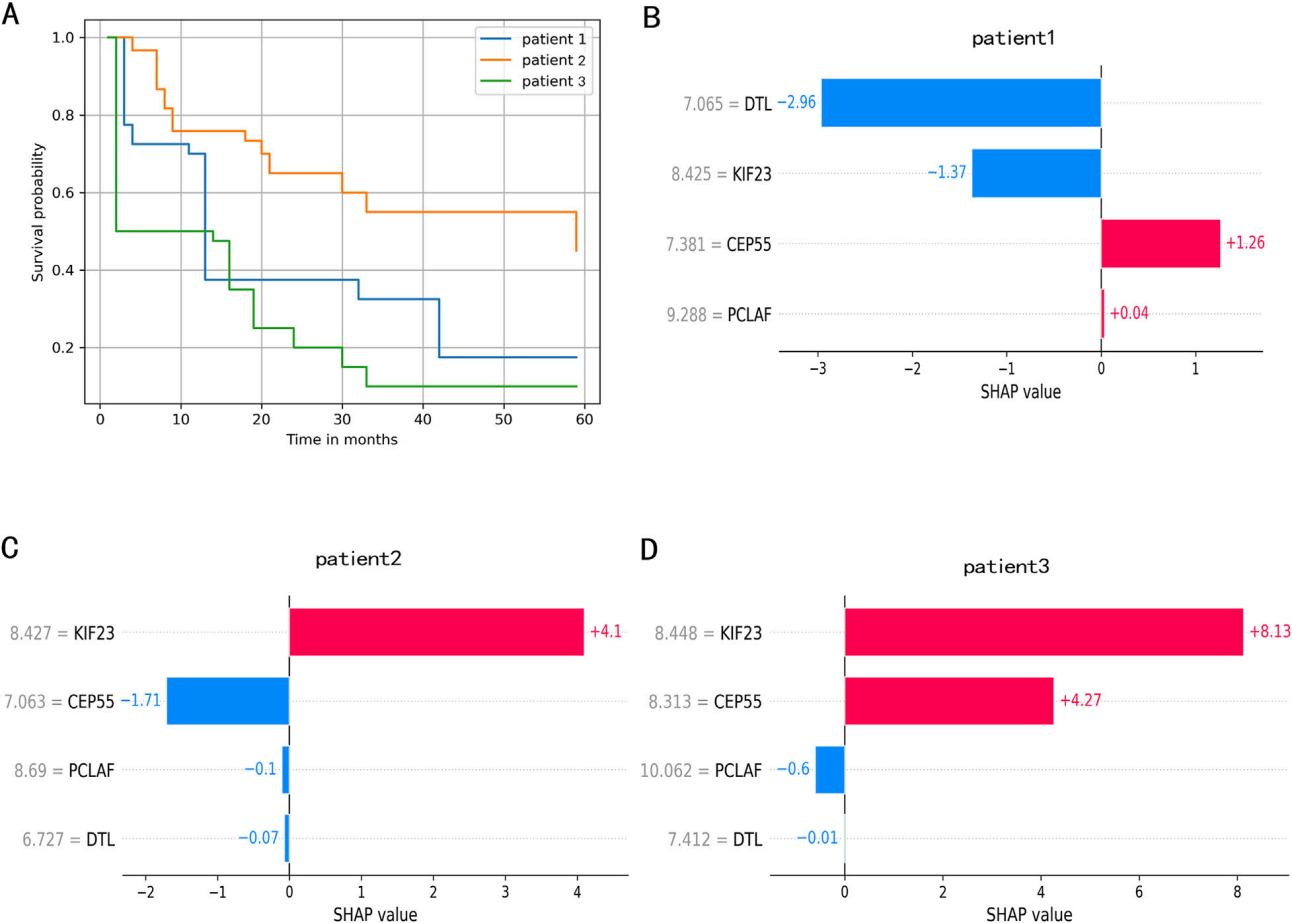
The individual survival prediction. **(A)** The estimated survival function of IPF patients. **(B)**. The individualized SHAP plot of the patient 1. **(C)** The individualized SHAP plot of the patient 2. **(D)** The individualized SHAP plot of the patient 3. In the individualized SHAP plot, red bands represent risk factors and promote poor prognosis, while blue bands are relative protective factors.

We randomly selected three patients to demonstrate individual survival prediction using the RSF model. Patient1: Expression levels of DTL, CEP55, PCLAF, and KIF23 were 6.727230, 7.063076, 8.690206, and 8.427149. Patient2: Expression levels of DTL, CEP55, PCLAF, and KIF23 were 7.064931, 7.381497, 9.288345, and 8.425116. Patient3: Expression levels of DTL, DTL, PCLAF, and KIF23 were 7.411715, 8.312722, 10.061563, and 8.447688. The individual predicted outcomes for these patients are shown in Figure 6. The personalized KM survival plots (Figure 6A) illustrate the survival probability for each individual at specific time points, while the individualized SHAP plots (Figures 6B–D) show the contribution of gene expression levels to each patient’s prognosis.

To enable clinicians to use the RSF model and the prediction tools, relevant files of the model have been uploaded to^2^. It provides a quicker and more intuitive way of predicting.

### GSEA enrichment analysis

The GSEA revealed that 32 pathways were significantly enriched in IPF compared to normal samples. In the high-risk group, 24 pathways were identified as enriched. After intersection analysis, we identified 7 pathways that were consistently enriched across both comparisons. The most significantly enriched pathways in the high-risk group included ECM receptor interaction, the MAPK signaling pathway, and focal adhesion, as illustrated in Supplementary Figure S4D.

### Immune function analysis

**FIGURE 7:**
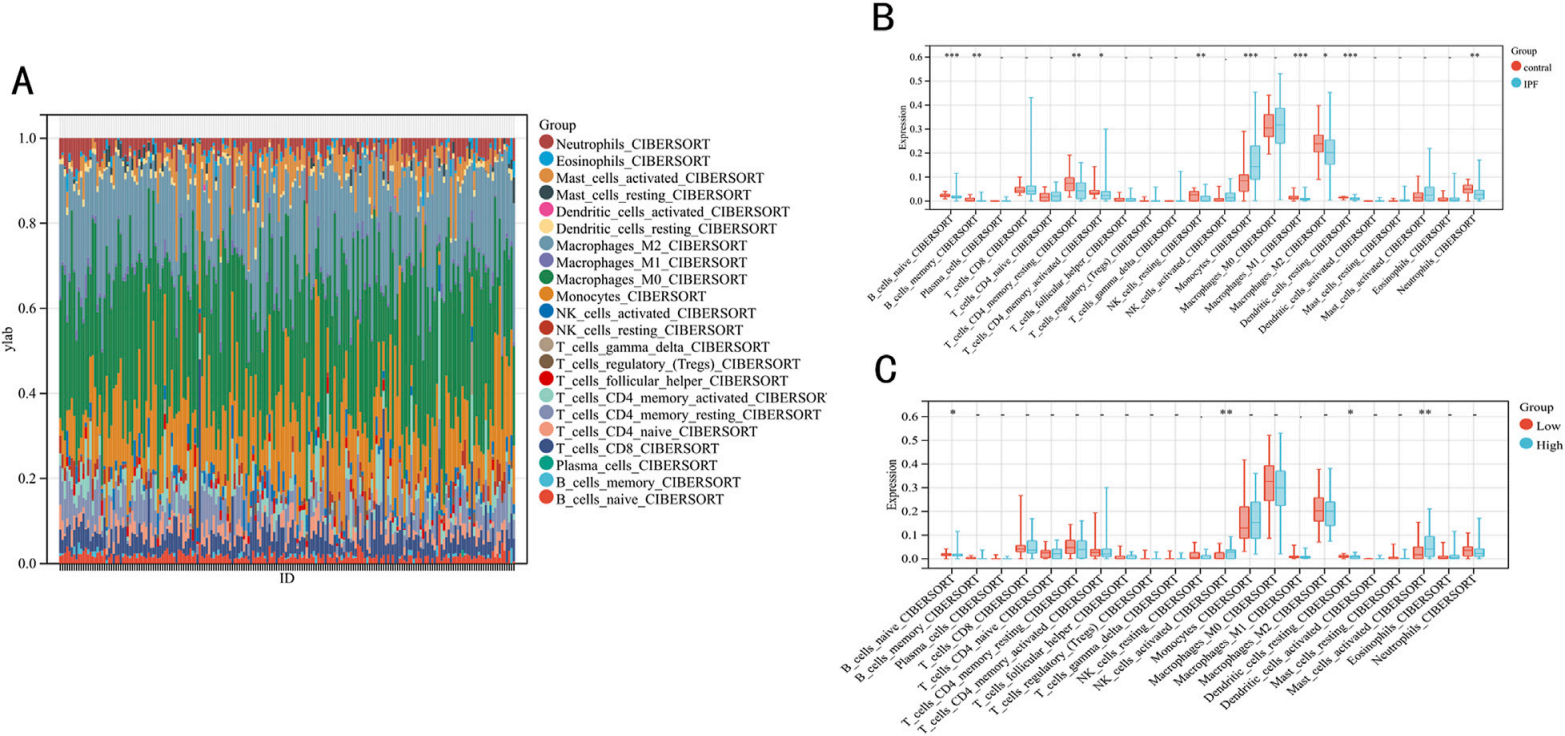
Immune infiltration between IPF and contral samples. **(A)** The relative percentage of 22 immune cells in each sample. **(B)** Differences in immune infiltration between IPF and contral samples. **(C)** Differences in immune infiltration between low-risk and high-risk groups.

To evaluate the impact of immune function on IPF, we employed the CIBERSORT algorithm to analyze differences in 22 types of infiltrating immune cells between IPF and normal samples (Figures 7A, B). We observed that the levels of B cell memory, B cells naive, T cells CD4 memory resting, T cells CD4 memory activated, NK cells resting, M1 macrophages, M2 macrophages, dendritic cells resting and neutrophils in the IPF were lower than those health samples, while the opposite was true for monocytes. Further analysis of the immune function of these differentially expressed immune cells within low-risk and high-risk groups revealed that the infiltration level of B cell naive (P < 0.02) and dendritic cells resting (P < 0.03) were significantly higher in the low-risk group than in the high-risk group (Figure 7C).

### Validation of the PF model

**FIGURE 8:**
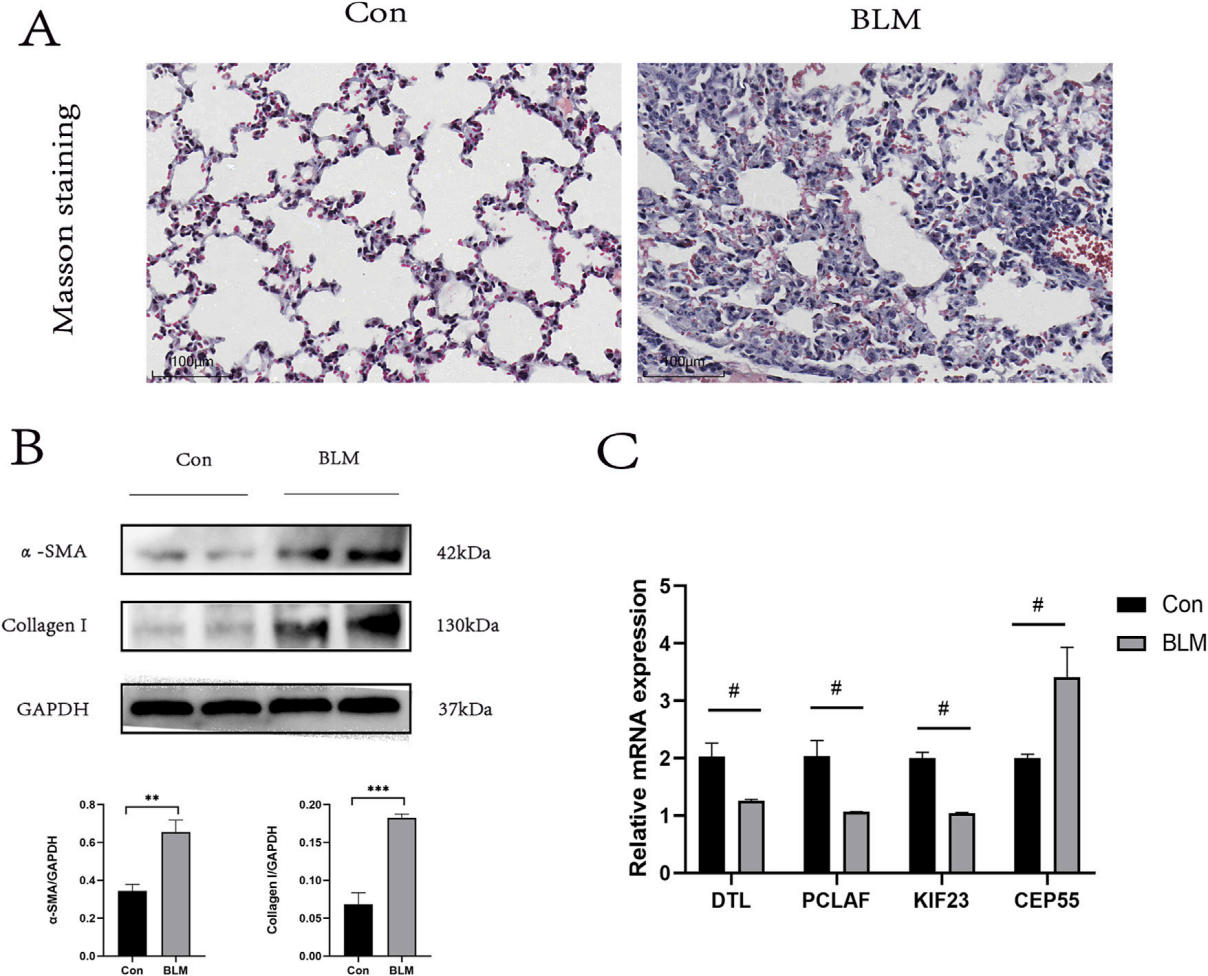
PF model sample validation. **(A)** Results of Masson staining, scale bar 100 μm. **(B)** In the lung tissue of PF mice protein levels of α- SAM, Collagen I (n = 3). **(C)** Expression levels of DTL, PCLAF, KIF23, and CEP55 were quantified using qRT-PCR analysis in lung tissue. ∗< 0.05, ∗∗< 0.01, ∗∗∗P < 0.001 by t-test. #p < 0.05 versus Con, t-test was used in **(C)**.

Given the challenges in acquiring clinical samples from IPF patients, we endeavored to validate the four hub genes identified in this study using a PF mouse model. Given the challenges in acquiring clinical samples from IPF patients, we endeavored to validate the four hub genes identified in this study using a PF mouse model [22]. The successful construction of the PF mouse model was confirmed by Masson’s trichrome staining and Western blot experiments (Figures 8A, B). RT-PCR was used to validate the expression of the hub genes incorporated into the RSF model and the results showed general agreement with the bioinformatics results (Figure 8C).

## Discussion

IPF is a chronic and progressive interstitial lung disease characterized by a poor prognosis, and there is a current deficiency in specific treatments [1–3]. The clinical symptoms of IPF are nonspecific, complicating the assessment of disease progression and prognosis based on clinical manifestations and auxiliary examinations alone. Therefore, identifying biomarkers and establishing a prognostic prediction model for IPF patients at the molecular level could facilitate individualized treatment of IPF patients, and benefit clinicians for the management of IPF patients.

In this study, we used transcriptional profiling data of bronchoalveolar lavage fluid from IPF patients to explore the correlation between biomarker levels and patient prognosis. First, 22 hub genes were identified, meanwhile, a Cox model and an RSF model based on an ML algorithm were established, and the results indicated that the RSF model had better predictive performance. The RSF model was then used to establish a new prognostic staging system, which was able to discriminate IPF patients into high-risk and low-risk groups, and the KM curve showed that this prognostic staging system had better discriminatory power. Finally, to gain further insight into the molecular mechanisms underlying the high-risk group in IPF, we used GSEA enrichment analysis to identify their main enriched pathways and used the CIBERSORT algorithm to determine the level of immune cell infiltration in the high-risk group.

Differently expressed 22 hub genes were obtained between IPF and normal samples from the GSE70866 of the GEO dataset by the different analysis of the limma and WGCNA module methods. The 22 hub genes in IPF were all higher expressed than normal samples. Through the GO enrichment analysis, we found the 22 hub genes were significantly enriched in the cell cycle. In particular, KEGG enrichment analysis showed that the 22 hub genes were significantly enriched in p53 signaling pathway, FoxO signaling pathway, cellular senescence, and cell cycle. The p53 signaling pathway and FoxO signaling pathway have been proven to be closely related to the development of fibrosis. Huang et al. reported that the p53 signaling pathway could affect the EMT progression of silica-induced pulmonary fibrosis [23]. Wang et al. had a similar conclusion in the model of renal interstitial fibrosis induced by unilateral ureteral obstruction [24]. Ma et al. reported the role of FoxO3a signal pathway in pulmonary fibrosis [25]. The review by Parimon et al. systematically expounded on the regulatory role of cellular senescence in pulmonary fibrosis [26]. Lv et al. confirmed that cell cycle inhibitor P21 promotes the development of pulmonary fibrosis by suppressing lung alveolar regeneration, and further confirmed that cell cycle is involved in the process of fibrosis [24]. These studies further substantiate the relevance of the 22 identified hub genes to fibrogenesis and disease progression.

On the basis of 22 hub genes, four prognostic genes (DTL,CEP55,PCLAF,KIF23) were further identified. KIF23, a microtubule-associated movement protein, is crucial in mitosis and cytokinesis [27]. Chen et al. reported that the downregulation of MiR-17-5p alleviates renal fibrosis by targeting KIF23 [28], which is consistent with the role of our RSF model, indicating that KIF23 may be a potential protective factor in the process of pulmonary fibrosis. The autophagy gene Cep55 is associated with bleomycin-induced pulmonary fibrosis [29]. The model established in this study suggests that a reduction in CEP55 expression is beneficial to the survival of patients with IPF. Similarly, DTL and PCLAF were also considered potential protective factors in the process of pulmonary fibrosis in our study.

To develop a predictive model with enhanced accuracy and clinical relevance, we employed various algorithms to construct the model, selecting the one that demonstrated superior predictive capabilities. We compared the traditional model (Cox algorithm) and the RSF model (ML algorithm), and the results showed that the RSF model based on the ML algorithm had better prediction performance. Given the unique requirements of the medical field, high predictive performance alone is insufficient to guarantee clinical utility; thus, we sought an effective method to elucidate the relationship between model variables and outcomes. The “black box” nature of ML-based models, which obscures the interpretation of results, has been a significant barrier to their clinical application [30]. To address this, we used the SHAP algorithm to render our model interpretable [31, 32], thereby clarifying the contribution of each variable to the outcomes and enhancing the model’s clinical utility. This high-performing prediction model facilitates the establishment of a more refined prognostic staging system for IPF patients, enabling more precise risk stratification and supporting the development of personalized treatment strategies. The RSF model has been refined into a user-friendly tool for individual survival prediction, providing risk scores, personalized Kaplan-Meier survival curves, and individualized SHAP plots. These features render individual predictions more intuitive and precise. In conclusion, we anticipate that this predictive tool will aid clinicians in evaluating patient prognoses to formulate tailored treatment plans. Furthermore, by quantifying individual prognosis risk levels, it is expected to enhance communication between clinicians and patients, thereby bolstering patient acceptance of prognostic information and treatment plans.

Patients with IPF could be divided into high-risk and low-risk groups based on prognostic staging systems. To deepen our understanding of the molecular pathways enriched in individuals at high risk for IPF, we performed GSEA on IPF patients and normal samples, as well as on high-risk and low-risk groups. This analysis identified seven significantly enriched pathways. Notably, ECM receptor interactions and MAPK signaling pathways have been reported to be closely associated with the development of pulmonary fibrosis. Han et al reported the role of ECM receptor interaction pathway in pulmonary fibrosis [33]. TGF-βs can regulate fibrosis via both canonical and non-canonical signaling pathways [34]. MAPK signaling pathway, as one of the non-canonical (non-Smad) signaling pathways, has demonstrated its role in pulmonary fibrosis [35]. Further exploration targeting these enriched pathways may be one of the directions to improve the prognosis of IPF.

Multiple studies have underscored the pivotal role of immunity in pulmonary diseases, including IPF [36]. Immune dysregulation is considered to be one of the bases of chronic lung diseases, including IPF [37]. Utilizing the CIBERSORT algorithm, we analyzed RNA-sequencing data to assess immune cell expression levels and derived the proportions of various immune cells within samples [38]. In this study, CIBERSORT was used to determine the level of immune cell infiltration between IPF and normal samples, as well as between high-risk and low-risk groups, and to determine two kinds of immune cells that may be related to the prognosis of high-risk IPF group, including B cell naive and dendritic cells resting. These two kinds of immune cell infiltration levels are lower in the high-risk group than in the low-risk group, indicating that they may be protective factors affecting the prognosis of pulmonary fibrosis.

Our research also has some limitations. First, the RSF model was established according to the GEO database, therefore, in order to validate our model, further clinical prospective studies are necessary. Second, functional experiments are needed to further reveal the potential mechanisms of hub genes in the future.

This study aims to establish a high-performance, clinically valuable prognostic prediction model at the molecular level, while also developing a new prognostic staging system that can understand the individualized prognosis of IPF patients and early identification of high-risk individuals. The study is based on the GSE70866 dataset, which has been reported in multiple studies, demonstrating its representativeness in IPF [39–43]. To establish a prognostic prediction model with universal applicability, unlike other previous studies that focused only on certain biological processes related to IPF, such as endoplasmic reticulum stress, autophagy, and immune-related genes [40, 42, 43], this study starts with all genes in IPF samples, aiming to screen out the most representative hub differential genes in IPF to establish a model. In addition, by constructing both traditional linear models and machine learning algorithm-based RSF models, the model with the best predictive performance was selected. Therefore, this study differs from currently reported studies in both variable selection and model algorithms. At the same time, the molecular mechanisms of high-risk individuals were further explored. Our findings provide a new and better tool for guiding individualized therapy in IPF and also provide new insights at a molecular level for improving the prognosis of IPF.

## Data Availability

Publicly available datasets were analyzed in this study. This data can be found here: All datasets used in the research can be found in Gene Expression Omnibus (https://www.ncbi.nlm.nih.gov/geo/, containing dataset of GSE70866).

## Footnotes

1
http://CIBERSORT.stanford.edu/

2
https://github.com/Renrende0328/IPF-RSFmodel
