# Supplementary material for: Integrating machine learning with bioinformatics for predicting idiopathic pulmonary fibrosis prognosis: developing an individualized clinical prediction tool
Source: Exp Biol Med (Maywood). 2024 Dec 23;249:10215. doi: 10.3389/ebm.2024.10215 (PMC11702306; doi:10.3389/ebm.2024.10215)
Supplement: Supplementary file 1 [file DataSheet1.docx]

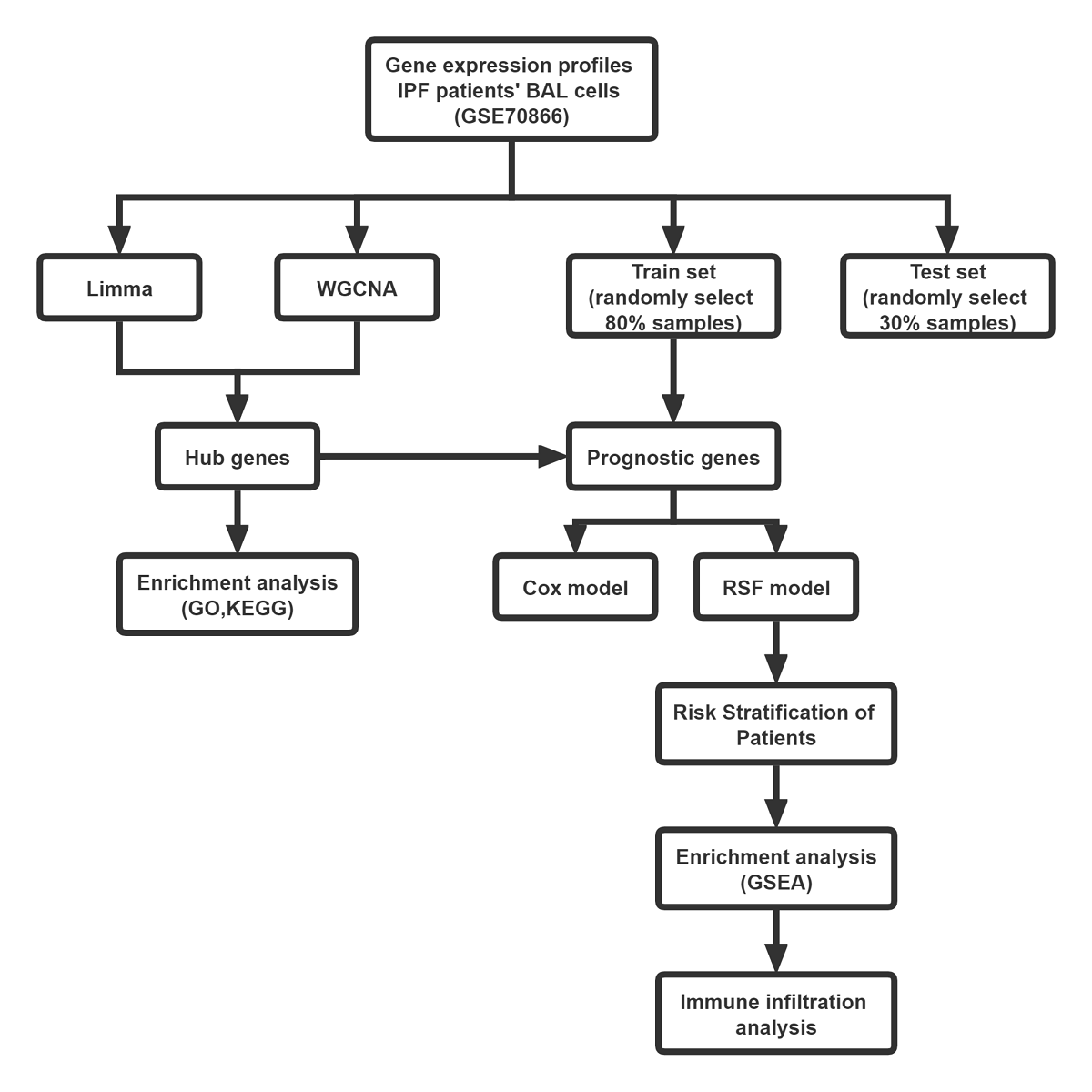


**Figure S1.** Flow chart of this study.


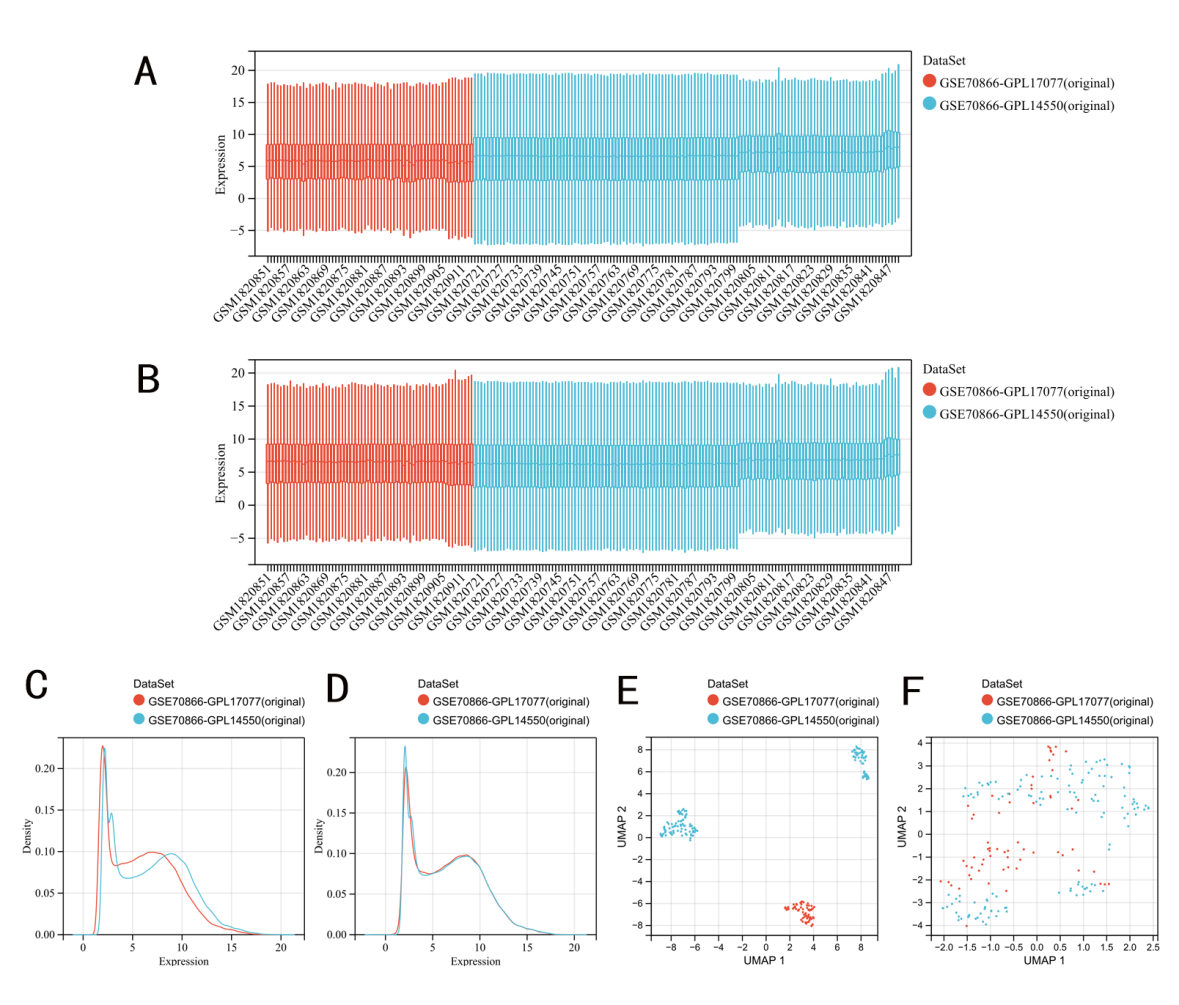


**Figure S2.** mRNA expression data of GSE70866 was merged. (A) (C) and (E) Before merger. (B) (D) and (F) After merger.


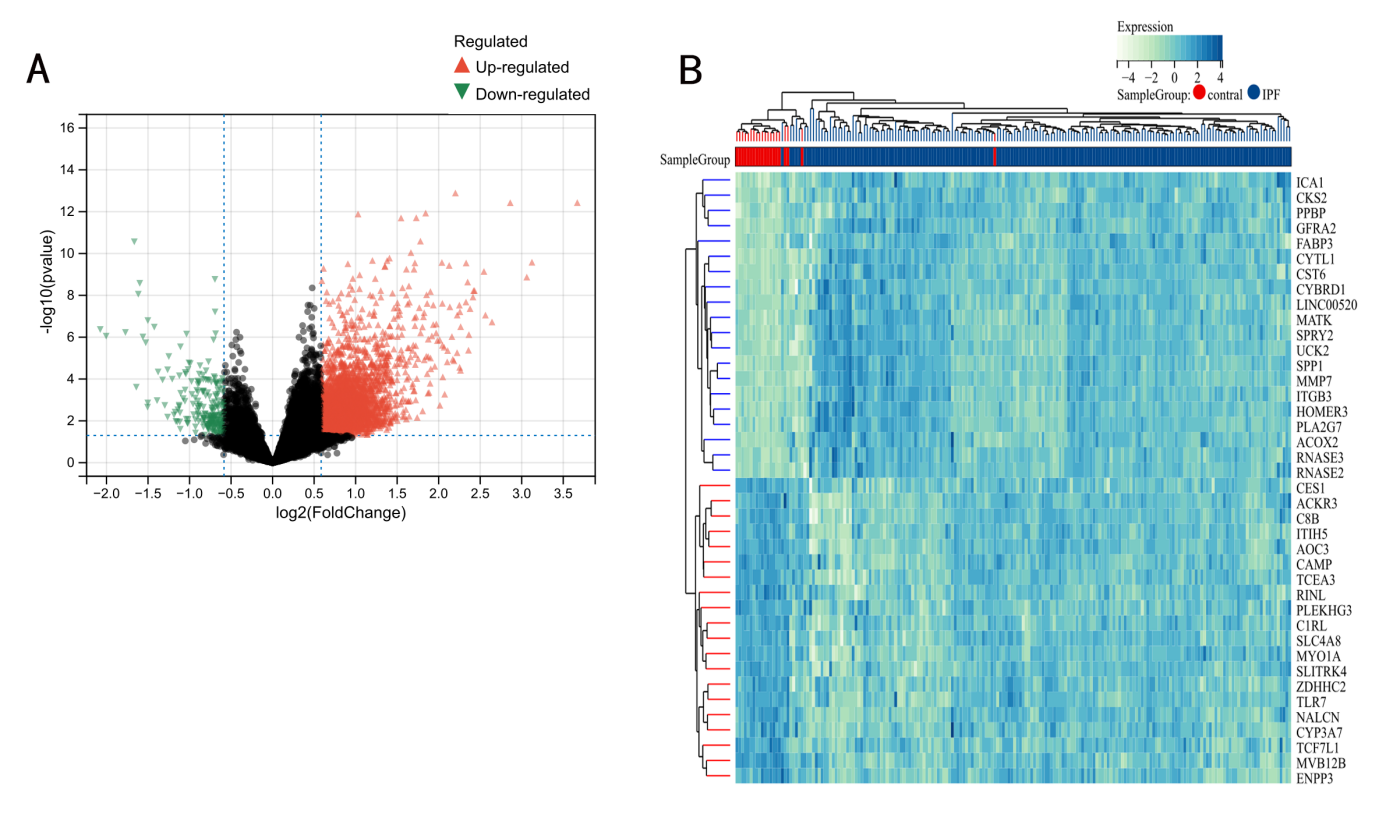


**Figure S3.** DEGs between IPF and contral samples. (A) Red genes represent significantly high expression in IPF, green genes represent significantly low expression in IPF, and gray genes indicate insignificant changes. (B) Heatmap of differentially expressed genes in IPF and controls.


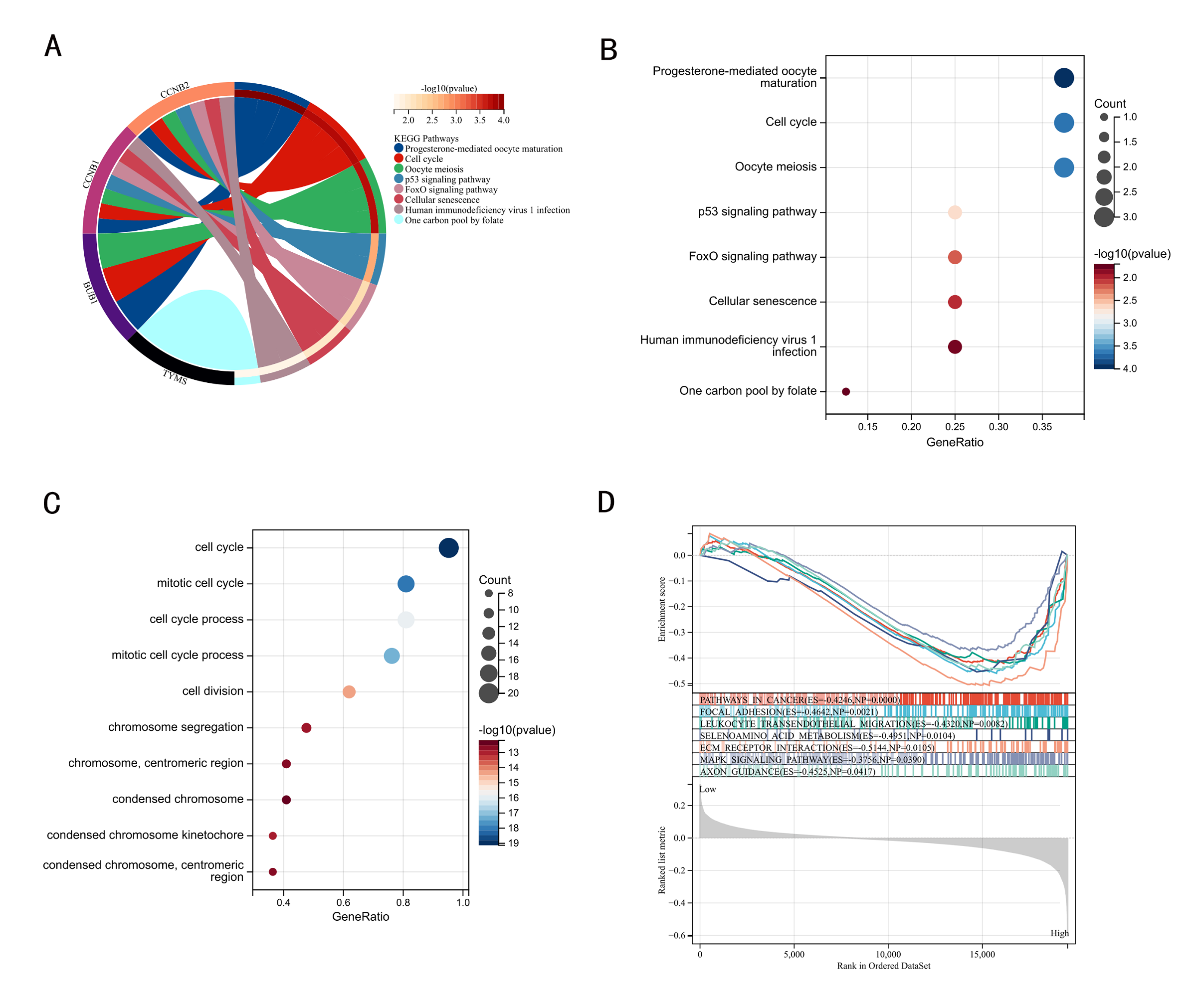


**Figure S4.** Functional enrichment analysis. (A) and (B) Biological processes in which the hub genes were involved. (C) The enriched GO terms of hub genes. (D) GSEA enrichment analysis between high-risk and low-risk groups.
